# Comparative Study of the Safety and Efficacy of a Prophylactic Insertion of Dr. Burke’s Every Second Matters-Uterine Balloon Tamponade (ESM-UBT) With IM Oxytocin vs. Only IM Oxytocin for the Prevention of Atonic PPH: A Randomized Parallel Group Trial

**DOI:** 10.7759/cureus.82052

**Published:** 2025-04-10

**Authors:** D Santhoshi, Rajasri G Yaliwal, Neelamma Patil, Aruna Biradar

**Affiliations:** 1 Obstetrics and Gynecology, Shri BM Patil Medical College, Hospital and Research Centre, Vijayapura, IND

**Keywords:** blood loss, brass v drape, high-risk pregnancy, postpartum hemorrhage, uterine atony, uterine balloon tamponade

## Abstract

Background

Uterine balloon tamponade (UBT) has emerged as a minimally invasive and cost-effective technique for managing atonic postpartum hemorrhage (PPH). This study assesses the safety and efficacy of prophylactic Dr. Burke’s Every Second Matters-UBT (ESM-UBT) combined with intramuscular (IM) oxytocin compared to IM oxytocin alone in preventing atonic PPH in high-risk women.

Methods

This randomized parallel-group trial was conducted over 1.5 years at Shri BM Patil Medical College, Hospital, and Research Centre, Vijayapura, Karnataka. A total of 226 women with high-risk pregnancies for atonic PPH were enrolled and randomized into two groups: Group 1 received prophylactic ESM-UBT with IM oxytocin (10 units), while Group 2 received only IM oxytocin. Blood loss was measured using the Brass V drape (Microtrack Surgicals Co., Ahmedabad, Gujarat, India) and additional gauze pad weight assessment. Hemoglobin levels were recorded pre-delivery and 48 hours postpartum. Statistical analysis was performed using IBM SPSS Statistics for Windows, Version 20.0 (IBM Corp., Armonk, NY).

Results

The prophylactic use of ESM-UBT significantly reduced total blood loss (Mean: 198.7 mL vs. 325.2 mL, p= 0.000001) and post-delivery hemoglobin drop (Group 1: 9.9 g/dL vs. Group 2: 9.2 g/dL, p = 0.0049). Blood loss at 5, 10, and 60 minutes postpartum was consistently lower in the ESM-UBT group (p 0.0001). The additional uterotonics and blood transfusion requirement was significantly higher in the IM oxytocin-only group (p = 0.003, p = 0.013, respectively).

Conclusion

Prophylactic ESM-UBT significantly reduces blood loss and the need for additional interventions in high-risk women, demonstrating its potential as a vital tool in PPH prevention, particularly in resource-limited settings. Its cost-effectiveness and ease of use make it a feasible global solution for improving maternal outcomes.

## Introduction

According to the World Health Organization (WHO), postpartum hemorrhage (PPH) is the leading cause of maternal mortality, especially in developing countries, accounting for about 127,000 maternal deaths per year [1]. A significant percentage of maternal deaths in low- and middle-income countries (LMICs) are caused by primary PPH, which is defined as blood loss exceeding 500 mL after a vaginal delivery or 1,000 mL after a cesarean delivery that occurs within 24 hours of delivery. Uterine atony is the most prevalent cause of PPH, accounting for almost 80% of primary PPH cases [1]. When the uterine muscles don’t contract properly after giving birth, it causes uterine atony, which results in excessive blood loss. Retained placental tissues, consumptive coagulopathy, uterine rupture, and lower genital tract trauma are additional essential causes of PPH.

Despite the widespread availability of uterotonic agents and advancements in obstetric care, PPH continues to pose a serious public health concern, especially in settings with limited resources where access to advanced interventions and skilled care is limited [2].

Timely intervention is essential to effectively treat PPH. The first-line treatment is still uterotonics, such as oxytocin, but in cases where these treatments are ineffective, further measures are required. When uterotonics or manual uterine massage are ineffective for atonic PPH, uterine balloon tamponade (UBT) has become a life-saving method. To apply pressure to the endometrial lining and myometrium to compress the blood vessels and achieve hemostasis, UBT entails inserting an inflatable balloon into the uterus [3]. As it avoids the need for more invasive procedures like uterine compression sutures, pelvic vessel ligation, arterial embolization, or hysterectomy (all of which require high skills and resource requirements), this minimally invasive approach has gained popularity [4].

Dr. Burke’s Every Second Matters-UBT (ESM-UBT) has become a popular and affordable choice among UBT devices. Launched for large-scale production in 2021, this one-time-use device was specifically created to overcome the obstacles that LMICs encounter when implementing traditional UBT solutions, like the Bakri balloon [5]. Healthcare professionals need little training to use the ESM-UBT, which is also much more user-friendly and reasonably priced. It is a useful and secure solution for managing PPH in environments with limited resources because of its design, which reduces the chance of cross-contamination [6].

PPH is still a leading cause of maternal morbidity, even with the availability of uterotonics, especially in environments with limited resources. According to recent research, UBT may be used as a supplement to lessen blood loss and the need for surgery. However, data on the preventative application of Dr. Burke’s ESM-UBT is still scarce. This study aims to determine whether prophylactic ESM-UBT insertion combined with intramuscular (IM) oxytocin lowers atonic PPH compared to IM oxytocin alone.

## Materials and methods

This randomized parallel-group trial was conducted over a period of 1.5 years at the Department of Obstetrics and Gynecology, BLDE (Deemed to be University) Shri BM Patil Medical College, Hospital, and Research Centre, Vijayapura, Karnataka. Women aged 18 years or older with high-risk pregnancies predisposing them to atonic PPH were recruited based on predefined inclusion and exclusion criteria. Written informed consent was obtained from all participants in accordance with the Declaration of Helsinki [7].

Ethical clearance to conduct the study was obtained from the Institutional Ethics Committee, BLDE (Deemed to be University), with reference number BLDE (DU)/IEC/911/2023-2024. The study was registered with the Clinical Trials of India (CTRI/2023/09/057173).

Sample size and statistical analysis

The total sample size is 226. With an anticipated proportion of total blood loss of more than 1000 mL in the UBT group at 79.6% and among controls at 52.5% [8], the study would require a minimum sample size of 113 per group (i.e., a total sample size of 226 assuming equal group sizes), to achieve a power of 99% at a two-sided significance level of p<0.05.

Data were analyzed using IBM SPSS Statistics for Windows, Version 20.0 (IBM Corp., Armonk, NY), and normality of data was tested before applying parametric tests. Results are expressed as mean ± standard deviation, median, interquartile ranges, counts, and percentages. Categorical variables are compared using the Chi-square test, and outcomes were analyzed using relative risk with corresponding 95% confidence intervals. A p-value < 0.05 was considered statistically significant.

Study design

Consenting women admitted for delivery with a diagnosis of high-risk pregnancy prone to atonic PPH were eligible for the study. The inclusion criteria included delivering viable babies in high-risk pregnancies predisposed to PPH, such as grand multiparity, hydramnios, pregnancy-induced hypertension, eclampsia, gestational diabetes, placental abruption, deranged coagulation profile, anemia, Rh incompatibility, and twin pregnancy. Exclusion criteria included cases of traumatic PPH, retained or adherent placenta, uterine rupture, anomalous uterus, suspected chorioamnionitis, purulent infections, and known advanced cervical cancer.

Randomization and group allocation

Simple randomization was performed using a computer-generated randomized program. Participants were randomized into two groups: (i) Group 1: Prophylactic insertion of Dr. Burke’s ESM-UBT with IM oxytocin (10 units) and (ii) Group 2: IM oxytocin (10 units) without the application of ESM-UBT.

Intervention and measurement

**Figure 1:**
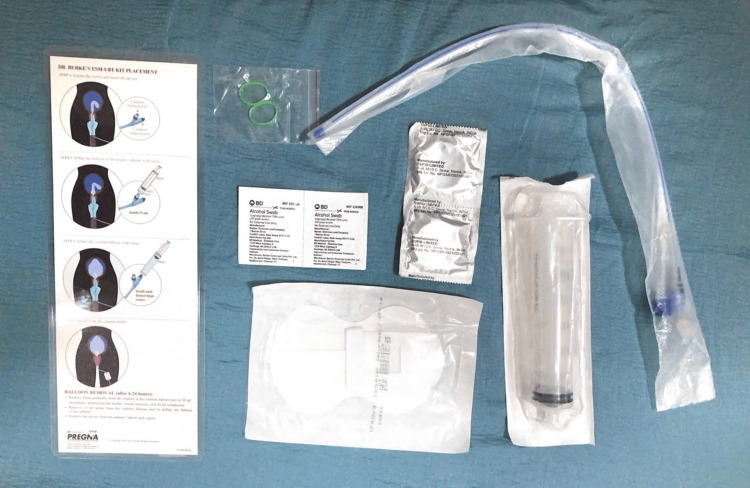
Dr. Burke's Every Second Matters-Uterine Balloon Tamponade (ESM-UBT) kit

The contents of Dr. Burke's ESM-UBT kit (Pregna International Ltd., Dabhel, Daman (U.T.), India) are as follows: 60 cc syringe, elastic O-ring, condom, antiseptic (providing iodine) prep pad, and Burke's catheter (Figure 1).

In Group 1, Dr. Burke's ESM-UBT is assembled by inserting the catheter halfway into a condom balloon. A silicone O-ring was wrapped four times around the base of the balloon to prevent saline leakage. The catheter was disinfected with iodine wipes before insertion into the uterus through the cervical opening. Gradual inflation was performed using normal saline (NS) with an initial 15 mL inflating the smaller catheter balloon and subsequent 50 mL inflating the main condom balloon via a one-way valve. Inflation continued until bleeding ceased, with a maximum recommended volume of 500 mL. The device is kept in situ after the expulsion of the placenta until six hours postpartum or till bleeding stops.

**Figure 2:**
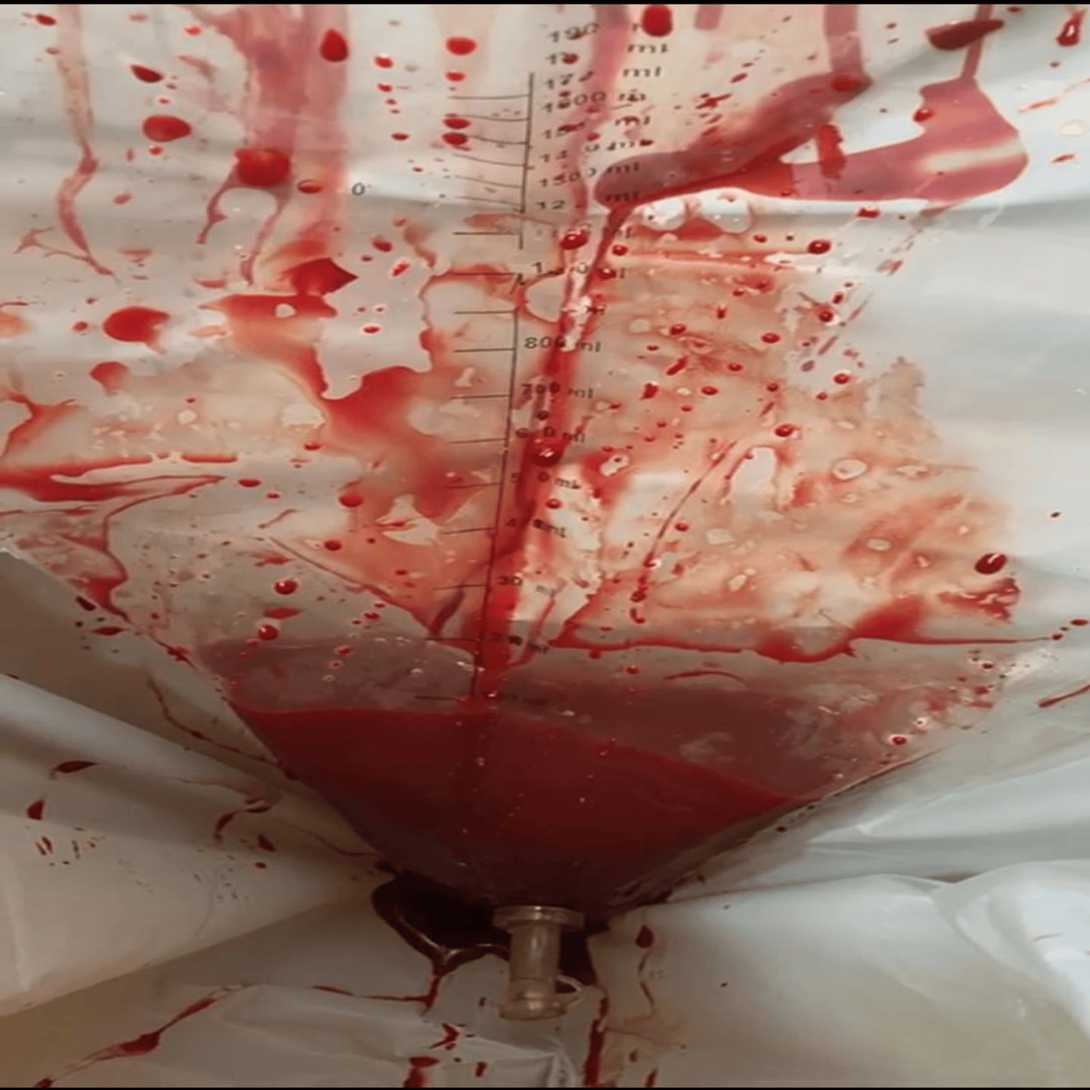
Blood loss measured in Brass V drape

Blood loss was measured using a calibrated Brass V drape (Microtrack Surgicals Co., Ahmedabad, Gujarat, India) (Figure 2). Additionally, gauze pads and clots were weighed to estimate total blood loss. Gauze mops are weighed before and after use, and the net amount of blood loss is recorded as approximately 1 gram, representing 1 milliliter of blood. Blood loss was measured and recorded in both groups. Hemoglobin (Hb) and white blood cell (WBC) counts were obtained pre-delivery and 48 hours postpartum using a Sysmex 1500 analyzer (Sysmex Corporation, Kobe, Japan).

## Results

**Figure 3:**
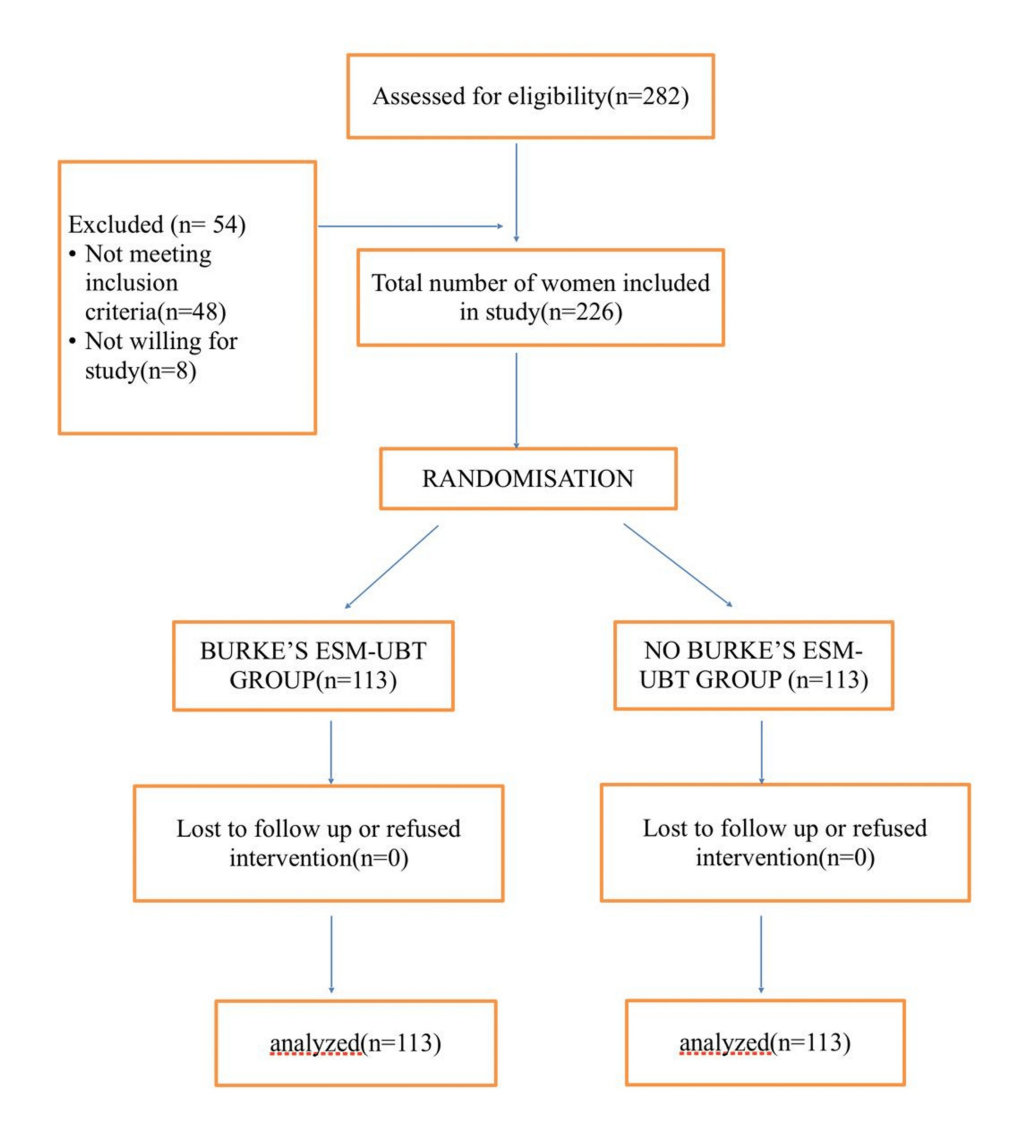
Consort flow chart ESM-UBT: Every Second Matters-Uterine Balloon Tamponade

A total of 282 women who underwent vaginal delivery during the study period were assessed. Of these, 54 were excluded as they did not meet the inclusion criteria. A total of 226 consenting women were included in the study and randomized into two groups as shown in (Figure 3).

The mean age of our study population was 29.5±4.9 years, 45% of the participants were between 38 weeks to 40 weeks of gestation at the time of enrollment, and risk factors among study groups were assessed. Based on the provided table comparing blood loss and hemoglobin (HB) levels between Group 1 (G1) and Group 2 (G2), significant differences were observed in most parameters. Total blood loss, including both drape and overall measurements, was significantly higher in Group 2 (P = 0.00001). Blood loss at 5, 10, and 60 minutes postpartum was also considerably more remarkable in Group 2, indicating a statistically significant (P-values ranging from 0.00001 to 0.0001). Hemoglobin levels before delivery were comparable between the groups (10.6 g/dL in Group 1 and 10.1 g/dL in Group 2 with P = 0.1108); however, post-delivery hemoglobin levels were significantly lower in Group 2, that is, 9.9 g/dL in Group 1 and 9.2 g/dL in Group 2 (P = 0.005). These findings suggest that Group 2 experienced more significant blood loss during and after delivery, leading to a more pronounced drop in hemoglobin levels. This data highlights the need for targeted interventions to minimize postpartum blood loss and preserve maternal health in Group 2 (Table 1).

**Table 1:** Comparative analysis of blood loss and hemoglobin levels between Group 1 (G1) and Group 2 (G2) HB: hemoglobin *Statistically significant

Variables	Mean	Standard deviation	95% CI	p-value	Test statistic
Blood in v drape (Group 1)	156.5	148.0	80-120	0.0000002*	t = -5.42
Blood in v drape (Group 2)	259.6	137.68	210-300		
Total blood loss (Group 1)	198.7	176.29	80-260	0.0000001*	t = -5.50
Total blood loss (Group 2)	325.2	164.46	261-364		
Blood loss just before delivery (Group 1)	24.6	21.46	10-20	0.0005945*	t = -3.45
Blood loss just before delivery (Group 2)	22.8	24.01	20-30		
Blood loss at 5 min after delivery (Group 1)	92.0	105.78	35.5-74.4	0.0000052*	t = -4.6
Blood loss at 5 min after delivery (Group 2)	155.7	98.64	110-178.7		
Blood loss at 10 min after delivery (Group 1)	51.9	45.83	30-50	0.0000011*	t = -5.0
Blood loss at 10 min after delivery (Group 2)	82.1	44.72	70-90		
Blood loss at 1 hour after delivery (Group 1)	30.6	20.75	20-30	0.0000000*	t = -5.85
Blood loss at 1 hour after delivery (Group 2)	49.6	27.05	40-50		
HB before delivery (Group 1)	10.6	2.05	10.2-10.9	0.1107970	t = 1.678
HB before delivery (Group 2)	10.1	1.85	9.7-10.4		
HB post-delivery (24 hrs - Group 1)	9.9	1.97	9.5-10.2	0.0048624*	t = 2.8
HB post-delivery (24 hrs - Group 2)	9.2	1.64	8.8-9.5		

The chi-square test results for the eight variables provide a mix of statistically significant and non-significant findings. For gestational diabetes mellitus (GDM), the risk ratio (RR) of 0.66 suggests a 34% lower likelihood of the outcome in Group 1 than in Group 2. Still, the p-value of 0.54 indicates this is not statistically significant. Similarly, anemia has an RR of 1.1, indicating a 10% higher likelihood of the outcome in Group 1 compared to Group 2, but the p-value of 0.50 shows no statistical significance. For pregnancy-induced hypertension and polyhydramnios, the RR of 1 indicates a similar outcome in Group 1 compared to Group 2, but the p-value of 1 indicates this is not statistically significant. Similarly, abruption and antepartum eclampsia have RR of 0.2, suggesting an 80% lower likelihood of an outcome in Group 1 compared to Group 2, but a p-value of 0.5 confirms no significant association. In twin pregnancy, an RR of 0.1 indicates a 90% lower likelihood of an outcome in Group 1 than in Group 2, but a p-value of 0.3 confirms no significant association. However, the use of additional uterotonics yielded an RR of 1.2, indicating a 20% higher likelihood of the outcome in Group 1, with a p-value of 0.002, demonstrating a statistically significant association (Table 2).

**Table 2:** Comparative analysis of blood loss and hemoglobin levels between Group 1 (G1) and Group 2 (G2) GDM: gestational diabetes mellitus, RR: risk ratio

S. No.	Variables	RR	95% CI	p-value	Chi-square value
1	Pregnancy-induced hypertension (for Group 1 and Group 2)	1	0.7-1.3	1	χ² = 0.0
2	Abruption (for Group 1 and Group 2)	0.8	0.4-1.6	0.5	χ² = 0.3
3	Antepartum eclampsia (for Group 1 and Group 2)	1.1	0.7-1.8	0.5	χ² = 0.5
4	Polyhydramnios (for Group 1 and Group 2)	1	0.2-4.0	1	χ² = 0.0
5	GDM (for Group 1 and Group 2)	0.7	0.3-1.5	0.54	χ² = 0.5
6	Twin pregnancy (for Group 1 and Group 2)	0.7	0.3-1.5	0.3	χ² = 0.8
7	Anemia (for Group 1 and Group 2)	0.9	0.6-1.1	0.50	χ² = 2.3
8	Any others (for Group 1 and Group 2)	1.1	0.7-1.9	0.59	χ² = 0.5

The use of additional uterotonics yielded an RR of 0.4, suggesting a 40% reduced risk of requiring additional uterotonics in Group 1 compared to Group 2. The 95% confidence interval (0.2 to 0.8) indicates that this result is statistically significant as it does not include 1 (the point of no effect). The p-value of 0.003 further supports that this result is statistically significant (p-value < 0.05), indicating that the intervention effectively reduces the need for additional uterotonics. Similarly, blood component transfusion showed an RR of 0.7, reflecting a 30% reduced risk of needing blood component transfusions in Group 1 compared to Group 2. The 95% confidence interval (0.5 to 0.9) suggests that this result is also statistically significant, as it does not include 1. The p-value of 0.013 supports the statistical significance of the result (p-value < 0.05), meaning the intervention has a significant effect on reducing the need for blood transfusions (Table 3).

**Table 3:** Comparative analysis of additional uterotonics and blood transfusion between Group 1 (G1) and Group 2 (G2) RR: risk ratio *Statistically significant

S. No.	Variables	RR	95% CI	p-value	Chi-square value
1.	Use of additional uterotonics	0.4	0.2-0.8	0.003*	χ² = 8.8627
2.	Blood component transfusion	0.7	0.5-0.9	0.013*	χ² = 5.5029

Thirty patients in the intervention group reported experiencing pain at the insertion site following UBT insertion. Additionally, spontaneous expulsion occurred in 34 patients within the intervention group.

## Discussion

According to the study, Dr. Burke’s ESM-UBT significantly reduces blood loss in women suffering from PPH. The intervention group consistently showed lower mean blood loss across various parameters, highlighting the effectiveness of this approach in reducing hemorrhagic complications after delivery.

By using Dr. Burke's ESM-UBT, the intervention group's mean blood loss in V drape was significantly lower at 156.5 (±176) ml than that of the control group, which was 325.2 (±164.4) ml. A p-value of less than 0.00002 indicated that the differences between the groups were statistically significant.

This notable distinction is consistent with the body of research that shows UBT to be a useful treatment for lowering blood loss in PPH cases. For instance, a study conducted by Babazhanova et al. (2022) to evaluate the efficacy of inexpensive uterine tamponade as a supplement to misoprostol for the treatment of uncontrolled postpartum hemorrhage revealed that a significantly higher percentage of women who received both tamponade and misoprostol than those who only received misoprostol experienced a total blood loss of more than 1000 mL, with a significant p-value (p=0.01) [9].

Likewise, the intervention group’s mean weight of blood-soaked gauze pads was significantly lower (41.4 (±34.0) g) than the control group’s (67.7 (±56.8) g) (p=0.001). These results support earlier studies that assessed 498 patients and showed that UBT helps lower total blood loss, which lowers transfusion rates and improves hemostasis [10].

The intervention group experienced a significantly lower total blood loss (mean: 198.7(±176.2)ml) than the control group (mean: 325.2(±164.4)ml) (p=0.000001). These results are consistent with systematic reviews emphasizing UBT as a conservative management approach that works well and lessens the need for other procedures like uterine artery embolization [11].

When evaluating blood loss at different postpartum time points, Dr. Burke's ESM-UBT demonstrated its efficacy. Compared to the control group, the intervention group showed noticeably less mean blood loss before delivery, five minutes, 10 minutes, and one hour after delivery. Previous clinical trials and systematic reviews that document the quick effectiveness of uterine balloon tamponade in reaching hemostasis within minutes of insertion corroborate these findings [12].

Clinical implications and significance

In settings with limited resources and no easy access to surgical interventions, uterine balloon tamponade provides a crucial, minimally invasive method of managing postpartum hemorrhage [13]. Furthermore, by reducing the incidence of severe anemia and the need for extensive transfusions, the use of UBT has been linked to lower maternal morbidity and mortality. This is consistent with a randomized controlled trial that was carried out in 2023 at the gynecological unit of the Mardan Hospital complex in Mardan, Pakistan, to examine 168 women who had suffered postpartum hemorrhage as a result of uterine atony following a vaginal birth [14]. With a significant P value of 0.006, the study concluded that uterine balloon tamponade was superior to uterine packing in reducing postpartum hemorrhage [14].

New evidence from recent studies

The use of UBT in the treatment of PPH has been further supported by recent research. According to a study by Khan and Malik (2023) conducted at the Mardan Medical Complex in Mardan, Pakistan, a randomized controlled trial showed that uterine balloon tamponade, with a success rate of 89.3% versus 72.6%, respectively, was significantly more effective than uterine gauze packing in reducing postpartum hemorrhage caused by uterine atony (p = 0.006) [14]. In China, a randomized controlled trial by Wei J et al. (2020) showed that intrauterine double-balloon catheterization significantly reduced maternal complications and blood loss compared to gauze packing (p < 0.01) in women with postpartum hemorrhage (PPH) after cesarean delivery for placenta previa. The catheterization achieved a high hemostasis success rate of 93.1% [15].

Regional and population-specific factors affecting outcomes

Regional and population-specific factors may be responsible for variations in UBT-related outcomes. The use of UBT is especially helpful in lowering maternal mortality in low-resource environments with limited access to surgical procedures and blood transfusion services. Cultural acceptance of specific medical interventions, provider training, and variations in healthcare infrastructure also influence the effectiveness of UBT. In high-income nations, the impact of UBT may be mitigated by access to sophisticated hemostatic procedures like uterine artery embolization, but research in sub-Saharan Africa shows that the availability of skilled medical professionals has a significant impact on the success of UBT application. Different results in managing PPH may also be caused by physiological and genetic factors, such as differences in coagulation profiles and the prevalence of anemia among various ethnic groups [9].

Mechanism of action

UBT has a unique mechanism for achieving hemostasis, which explains why the ESM-UBT group experienced significantly less blood loss than the oxytocin-only group. The ESM-UBT directly applies intrauterine pressure, promoting mechanical tamponade of bleeding vessels, in contrast to oxytocin, which primarily stimulates oxytocin receptors to induce myometrial contraction. By compressing the venous sinuses inside the uterine wall, this pressure effect helps to quickly and efficiently stop blood loss [9]. Furthermore, even when myometrial contraction is delayed, the uterus can maintain hemostasis because the tamponade effect stabilizes clot formation [12]. The prophylactic use of ESM-UBT resulted in significantly less blood loss in the study population, which can be explained by this dual-action strategy.

Furthermore, balloon tamponade achieves hemostasis in 80-90% of cases, frequently avoiding the need for additional surgical intervention, according to recent studies comparing it with oxytocin-alone management in atonic PPH [10]. Since myometrial dysfunction is the primary cause of atonic PPH, combining mechanical compression with pharmacologic contraction will probably achieve a better synergistic effect.

Cost-effectiveness of prophylactic UBT versus reactive PPH treatment

Even though the study shows that prophylactic UBT lowers the need for additional uterotonics and blood transfusions, more research is needed to determine how cost-effective it is in comparison to reactive PPH treatment. According to research, reactive UBT use for PPH management is cost-effective, particularly in low-resource environments with limited access to surgical procedures and blood transfusions [13]. However, the device's cost, the strain on the healthcare system, and the overall incidence of PPH all affect how cost-effective prophylactic application is. Using a low-cost UBT device for reactive PPH treatment significantly decreased maternal morbidity and mortality while remaining cost-effective when compared to transfusion-heavy management strategies, according to a study done in sub-Saharan Africa [13].

A direct economic analysis contrasting routine use of UBT with reactive treatment is required to ascertain whether the additional expense is warranted, even though prophylactic UBT may further reduce blood loss and the need for subsequent interventions. Future research should include cost-benefit analyses and long-term maternal health outcomes to determine whether prophylactic ESM-UBT is more cost-effective than reserving UBT for cases of active hemorrhage.

Study strengths

This study’s randomized controlled design is a major strength since it reduces bias and guarantees solid, trustworthy data for clinical decision-making. By distributing confounding variables among the groups in an even manner, randomization makes sure that any variations in results are due to the interventions under test. This methodological rigor improves the study's credibility and suitability for clinical practice.

In order to measure blood loss, a crucial component of managing PPH, the study also used calibrated tools such as the Brass V drape. Blood loss must be accurately measured to diagnose and treat PPH.

The Brass V drape is a dependable option for this investigation since it is a well-known instrument for accurately measuring blood loss, as evidenced by earlier studies. This precision guarantees the reliability of blood loss results, which is essential for evaluating the efficacy of the two interventions being studied.

Limitations of the study

The study has significant limitations despite its strengths. Research conducted in a single location limits the findings' applicability to larger and more varied populations. Although the results of the study support the efficacy of Dr. Burke's ESM-UBT, the study's single-center design, exclusion of cases requiring surgical procedures other than UBT, and lack of long-term follow-up on maternal outcomes are some possible drawbacks. Future multi-center studies evaluating the relative effectiveness of various balloon tamponade methods, including the Bakri balloon and condom catheter, are necessary [16].

## Conclusions

This study demonstrates that prophylactic ESM-UBT significantly reduces blood loss and the need for additional interventions. Consistent with existing literature, the device significantly minimizes total blood loss, need for additional uterotonics and decreases transfusion rates, making it a cost-effective and easy-to-use tool with minimal training requirements, especially in low-resource settings with high maternal mortality from hemorrhage. Future research should assess its cost-effectiveness, long-term maternal outcomes, and feasibility for widespread implementation in low-resource settings.
